# Chronic Alcohol Treatment-Induced GABA-Aα5 Histone H3K4 Trimethylation Upregulation Leads to Increased GABA-Aα5 Expression and Susceptibility to Alcohol Addiction in the Offspring of Wistar Rats

**DOI:** 10.3389/fpsyt.2018.00468

**Published:** 2018-10-24

**Authors:** Kuan Zeng, Aimin Xie, Xiaojie Zhang, Baoliang Zhong, Xuebing Liu, Wei Hao

**Affiliations:** ^1^Research Center for Psychological and Health Sciences, China University of Geosciences, Wuhan, China; ^2^Affiliated Wuhan Mental Health Center, Tongji Medical College of Huazhong University of Science & Technology, Wuhan, China; ^3^Key Laboratory of Psychiatry and Mental Health of Hunan Province, China National Clinical Research Center for Mental Health Disorders, Mental Health Institute of the Second Xiangya Hospital, National Technology Institute of Psychiatry, Central South University, Changsha, China

**Keywords:** alcohol, addiction, GABA-Aα5, histone H3K4 trimethylation, offspring

## Abstract

Gamma-aminobutyric acid (GABA)-Aα5 is considered to be associated with alcohol-induced memory deficits. However, whether it participates in the formation of alcohol addiction or in the regulation of its susceptibility is unknown. Here, we used a chronic alcohol treatment model to obtain alcohol-addicted Wistar rats. Long-term alcoholism increased the expression of prefrontal cortex GABA-Aα5 by inducing its histone H3K4 trimethylation, and these changes could be hereditary and lead to increased vulnerability to alcohol addiction in offspring. This study indicates the risk of long-term alcoholism in future generations, emphasizes the importance of GABA-Aα5 in the formation of alcohol addiction and the regulation of its susceptibility, and provides new evidence regarding the mechanisms underlying alcohol addiction.

## Introduction

Although it is a substance that elicits dependency, alcohol has legitimacy and widespread social acceptance. In 2015, a study showed that ~18.3% of adults experienced drunkenness 1 month before the investigation, and 843.2 per 100,000 people were suffering from alcohol addiction (1). The World Health Organization's global status report on alcohol and health in 2014 also indicated that in 2012, 3.3 million people worldwide died due to harmful alcohol use, and this number exceeded the total number of deaths caused by AIDS, tuberculosis, and violence, accounting for 5.9% of all deaths worldwide (2). However, owing to the unknown mechanism of alcohol addiction, there is a lack of curative and prophylactic treatments.

At present, research on the biological mechanism of mental addiction to addictive substances indicates that the dopamine (DA) system at the edge of the midbrain, which is also known as the “reward system,” plays a key role (3). This theory is consistent with the idea that “there is no addiction without rewards.” Gamma-aminobutyric acid (GABA) is the major inhibitory neurotransmitter in the central nervous system and can participate in reward response by inhibiting the DA system of the midbrain (4). In addition, in alcohol addiction, GABA (A) receptors are associated with alcohol tolerance, dependence, and withdrawal (5). As an important subunit of GABA-A, the GABA-Aα5 subunit is thought to be closely related to cognitive impairment. Lack of GABA-Aα5 receptors in mice induces spatial memory and learning impairment in a water maze (6). A significant increase in memory was observed in wild type mice treated with GABA-Aα5 selective inverse agonists (7). In a study of addictive behavior, the use of GABA-Aα5 inhibitors reduced alcohol-induced memory loss (8), while prenatal chronic alcohol exposure resulted in dysregulated expression of GABA-Aα5 and impaired learning and memory in offspring (9). Thus, GABA-Aα5 plays a very important role in the regulation of alcohol-dependent cognitive impairment. However, whether GABA-Aα5 is involved in the formation of alcohol addiction is unknown.

In this study, we used a chronic alcohol treatment model to ascertain the relationship between alcohol addiction and GABA-Aα5. We found that increased prefrontal cortex (PFC) GABA-Aα5 expression and its histone H3K4 trimethylation caused by chronic alcohol treatment could be inherited by the offspring, increasing the vulnerability of the offspring to alcohol addiction. Our results provide new evidence regarding the mechanisms of alcohol addiction.

## Materials and methods

### Animals and treatments

All Wistar rats were provided by the Henan animal experimental center. Laboratory animals were kept in special well-ventilated animal houses with a 12-h light cycle (8 a.m. to 8 p.m.) at a room temperature of 25 ± 0.5°C and ~55% humidity. Rats weighed 190–225 g at the beginning of the experiment, and 3–4 rats were housed in each cage (clean grade) with free access to food and water. Rats were allowed to adapt to the environment in the animal house 1 week before the experiment. For the first 3 days of the experiment, the operator touched and grasped the animals briefly (3 min/rat/day) to promote adaptation to this brief grip. All experiments were carried out during the day (8 a.m. to 8 p.m.). The experiment was conducted in accordance with the animal experiment regulations of Xiangya Medical School and Xinxiang Medical College of Central South University.

#### Parental generation

**Figure 1:**
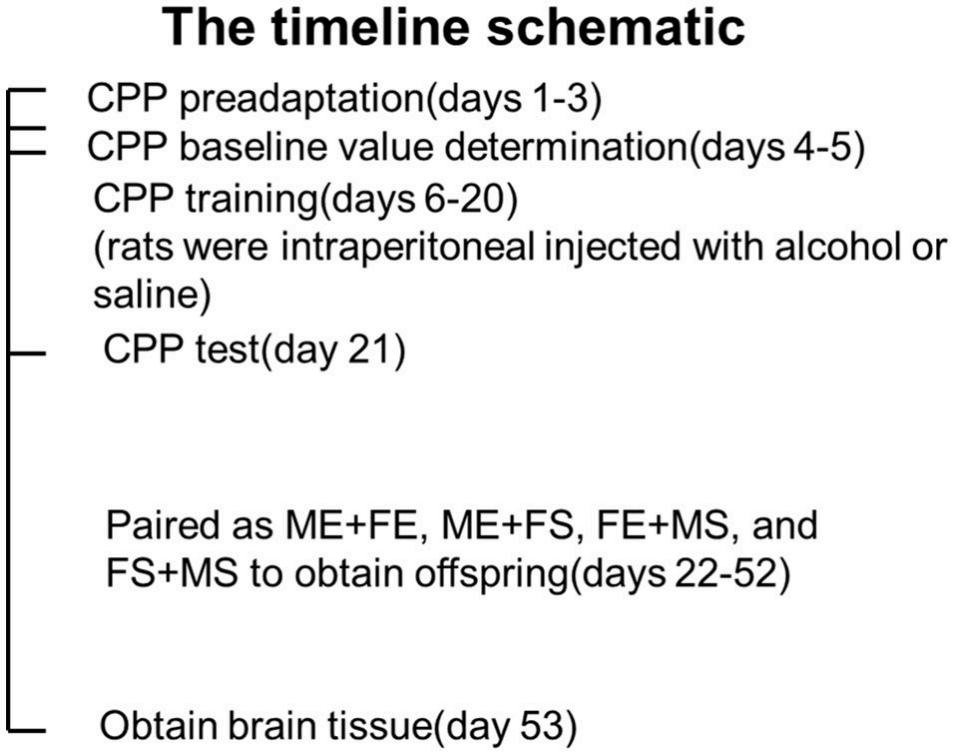
The timeline schematic of rats treatment.

A total of 24 Wistar rats, including 12 males and 12 females, were randomly divided into two groups of six rats for each gender as follows: male ethanol treatment group (ME), female ethanol treatment group (FE), male saline treatment group (MS), and female saline treatment group (FS). All rats were subjected to conditioned place preference (CPP) testing. However, for the ME and FE groups, pure alcohol was diluted to 10% alcohol with saline, and 0.5 g/kg was administered by intraperitoneal injection every day for 15 days during the training period. For the MS and FS groups, the same amount of saline was injected. The rats were paired as ME + FE, ME + FS, FE + MS, and FS + MS to obtain offspring, and they were sacrificed to obtain brain tissue 1 month later (Figure 1).

#### Offspring

All offspring were grouped by their parents as ME + FE (MEFE), ME + FS (MEFS), FE + MS (FEMS), and FS + MS (FSMS). At 2 months of age, six rats in each group were sacrificed to obtain brain tissue, and the remaining six were tested by CPP with chronic alcohol treatment during the training period.

### Chemicals

Anhydrous ethanol was purchased from Tianjin De En Chemical Reagent Co., Ltd. (Tianjin, China). Sodium pentobarbital was purchased from Sigma (USA).

### Conditioned place preference (CPP)

Rats were randomly divided into four groups of six animals as mentioned above. We used nonequilibrium training (convergence process). An automated computer monitoring system was used for records.

#### CPP preadaptation (days 1–3)

To promote adaptation to the experimental environment, rats were placed in a CPP box with free access to the black and white sides for a period of 60 min every day for 3 days.

#### CPP baseline value determination (days 4–5)

Rats were placed in the CPP box for 15 min, and the time spent on the black or white sides of the box was recorded. Detection is performed twice in a day. The mean of the baseline values of the two tests is the CPP baseline value(s), and the side on which the rats spent less time (< 450 s) was used as the side for CPP training with addictive drugs.

#### CPP training (days 6–20)

A clapboard was used to separate the CPP boxes. We placed 10% alcohol on the side of the CPP box for addictive drugs, and rats were trained for 60 min in the morning. Normal saline was placed on the other side of the CPP box, and rats were trained for another 60 min in the afternoon. The next day, the training sequence was reversed; this process was continued for 15 consecutive days.

#### CPP test (day 21)

Twenty-four hours after the last training, rats were placed in the box without the clapboard and with free access to the black and white sides for 15 min. The time spent on each side was recorded.

### Quantitative RT-PCR (real-time polymerase chain reaction)

Total RNA from brain tissue was extracted using TRIzol Reagent (Invitrogen, Waltham, MA, USA). First strand complementary DNA (cDNA) was synthesized from 500 ng total RNA using a high capacity cDNA reverse transcription kit (Applied Invitrogen, Shanghai). Quantitative polymerase chain reaction (PCR) was performed in a 10 μL standard PCR mixture prepared in duplicates using an Applied Biosystems 7900 Prism Real-Time PCR system and SYBR Premix Ex Taq (TaKaRa, Dalian, Japan) in accordance with the manufacturer's protocol. Quantitative PCR primers for GABA-Aα5 were as follows: 5′-AAGATGAAAGGCTGCGGTTTA-3′ and 5′-CCATGAGGTGGTACTGGTTGA-3′. PCR primers of GAPDH were 5′-ACGTAGCTCAGGCCTCTGCGCCCTT-3′ and 5′-CTGGCACTGCACAAGAAGATGCGGCTG-3′. The forward primer binding site is located on the sixth exon, and the reverse primer binding site is located on the ninth exon. The size of the PCR product is 372 bp, and the PCR cycling conditions were 95°C for 5 min, 95°C for 30 s, 60°C for 45 s, 72°C for 45 s, and 72°C for 5 min for 35 cycles. Subsequently, the products were maintained at 4°C. After the amplification reaction was completed, the solution curve was produced by gradually increasing the temperature and monitoring the fluorescence signal at each step to determine the response specificity. The cDNA amplification reaction was performed in the presence of SYBR Green in triplicates. The Ct value of each sample was obtained using the FTC-2000 software. To avoid differences in the initial total copy number due to differences in the amount of cDNA in each sample, we used the normal housekeeping gene *GAPDH* as an internal reference to normalize each sample. By using the delta-delta CT method, multiple changes in the mRNA levels and control values were calculated to compare the relative expression results among different treatments. The relative expression of mRNA in the experimental group compared with that in the control group was calculated using the 2^−ΔΔ*CT*^ method. ΔCT = CT (GABA-Aα5) – CT (GAPDH), ΔΔCT = ΔCT (experimental group) – ΔCT (control group).

### Chromatin immunoprecipitation (ChIP)

This procedure was performed based on the instructions of a chromatin immunoprecipitation (ChIP) kit (number 17-371, Millipore, Massachusetts, USA) with a few modifications. Frozen PFC tissue was sectioned into pieces and immediately cross-linked in formaldehyde for 10 min. Glycine was added to quench the cross-linking reaction, and the tissue was subsequently washed with PBS containing proteinase inhibitor, followed by chromatin extraction by SDS lysis buffer. With the optimal conditions for sonication, chromatin was sheared to 200–1,000 bp, concentrating on 400–500 bp. We centrifuged the homogenate and moved the supernatant to fresh microfuge tubes. The tubes were designated as positive control (anti-RNA Polymerase II), negative control (normal mouse IgG), and anti-acetylation; ChIP dilution buffer containing protease inhibitor was added to each tube to dilute the chromatin lysate. We precleared the chromatin solution with salmon sperm DNA/protein G agarose, followed by brief centrifugation. We removed 10μl of the supernatant as input for the purpose of performing normalization later. We collected the remaining supernatant for immunoprecipitation overnight at 4°C with antibodies directed against H3 acetylation on Lys9 (kit number 9671, Cell Signaling Technology), tri-methyl-H3 (Lys4) (kit number 9727, Cell Signaling Technology), RNA polymerase II, and normal mouse IgG. After immunoprecipitation, we collected the DNA-histone complex with salmon sperm DNA/protein G agarose beads. The beads were washed with low salt buffer, high salt buffer, LiCl buffer, and TE buffer. The DNA-histone complex was eluted from the beads with elution buffer in fresh tubes. All tubes including inputs and immunoprecipitates were incubated at 65°C for 5 h. RNase A was then added and incubated at 37°C for 30 min. Next, we added proteinase K, 0.5 M EDTA, and 1 M Tris-HCl for 1-h incubation at 45°C. The DNA associated with acetylated histones was purified and collected in elution buffer. Most ChIP experiments were performed twice on two independent tissue samples for confirmation.

### Statistical analysis

All data are presented as the mean ± SD. Two-way analysis of variance (ANOVA) followed by the least significant difference (LSD) as *post-hoc* test was used to analyze the data in all figures. *P* < 0.05 indicates a significant difference. All statistical analyses were performed using SPSS 19.0.

## Results

### Chronic alcohol treatment increases the conditioned place preference in rats

**Figure 2:**
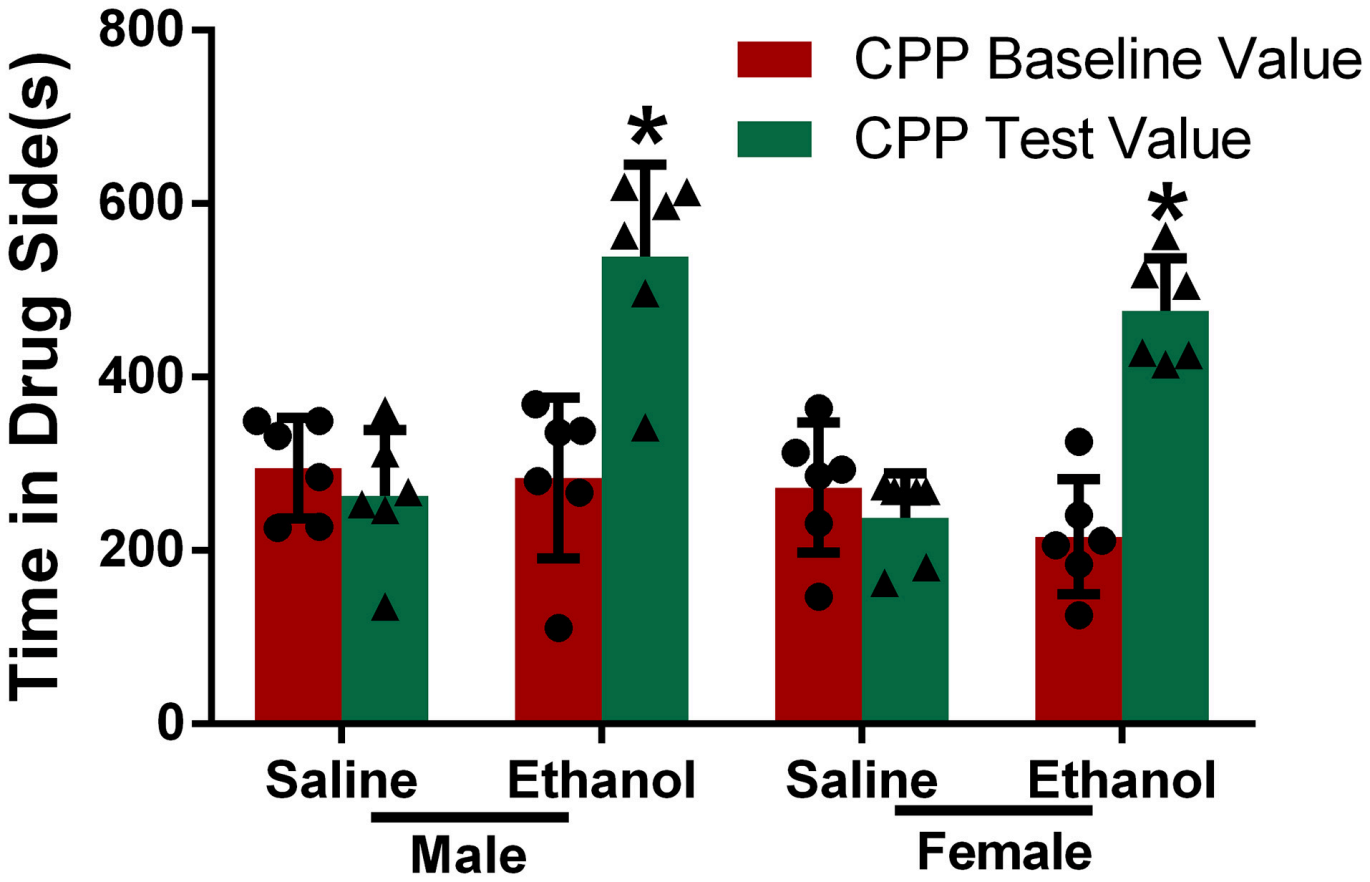
Chronic alcohol treatment increased the conditioned place preference in rats. Rats were divided into four groups. Rats injected with saline or ethanol were denoted as Saline or Ethanol, and each group was divided into male and female by gender. CPP baseline value and CPP test value were recorded. The data are expressed as the mean ± SD (*n* = 6). ^*^p < 0.05 vs. Saline, two-way ANOVA.

Conditioned place preference is often used to detect incentive motivation and can partly represent the degree of drug dependence (10). The results of the CPP test in the four groups of rats are shown in Figure 2. There was no significant difference in CPP baseline values between the four groups, and the values were comparable. The CPP test values were significantly higher than CPP baseline values after chronic alcohol treatment in both the male and female groups (*P* < 0.05), whereas there was no significant difference between the CPP test values and CPP baseline values in the groups treated with saline, indicating that the two groups of rats formed a clear CPP for alcohol.

### Chronic alcohol treatment increases GABA-Aα5 mRNA levels in the PFC

**Figure 3:**
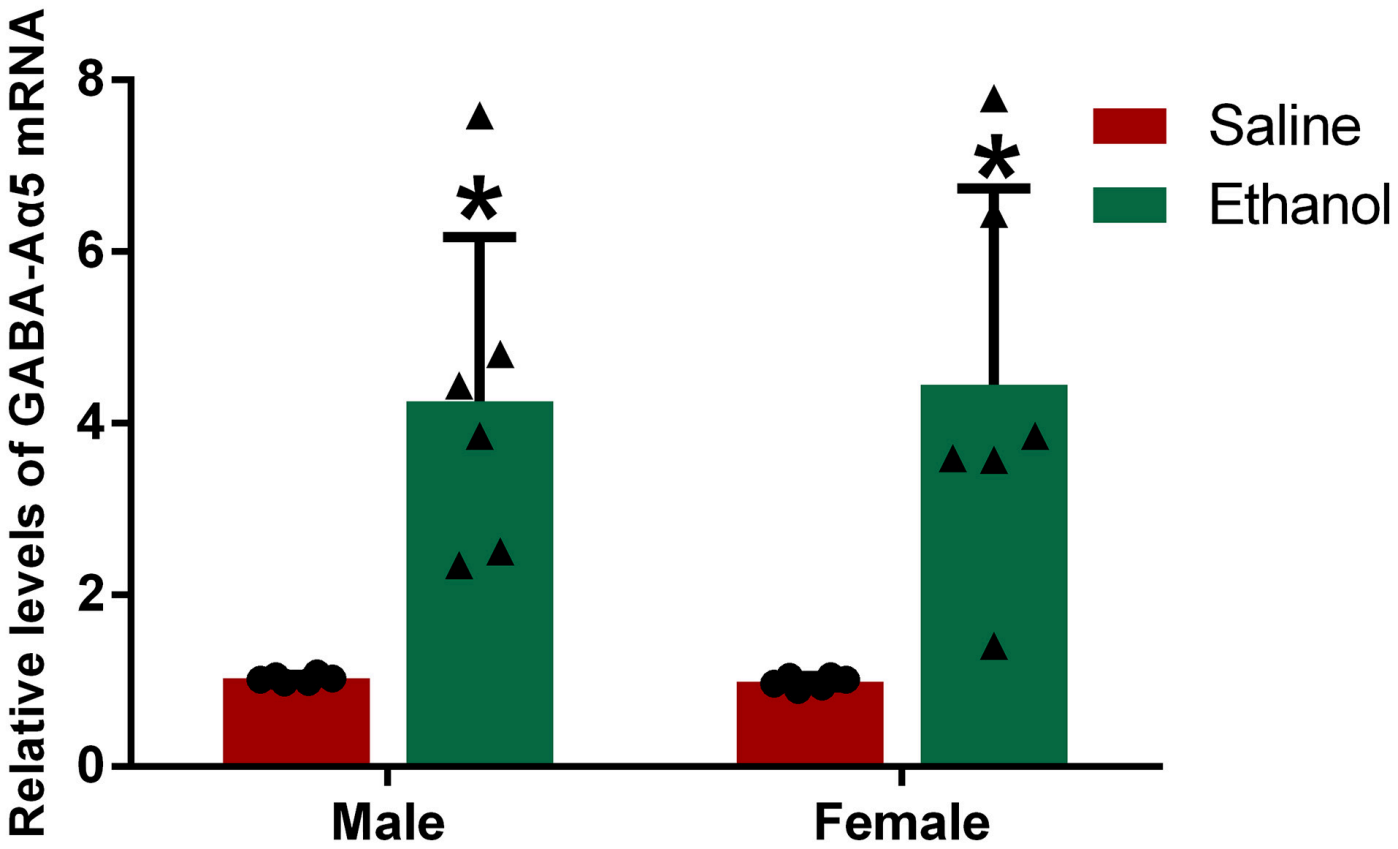
Chronic alcohol treatment increased GABA-Aα5 mRNA levels in the PFC. The relative level of GABA-Aα5 mRNA in the PFC was detected by RT-PCR. The data are expressed as the mean ± SD (*n* = 6). ^*^p < 0.05 vs. Saline, two-way ANOVA.

In Wistar rats, GABA-Aα5 was previously reported to be positively correlated with spontaneous alcohol consumption, but the relationship between GABA-Aα5 and alcohol addiction is unknown (5, 11). To investigate this relationship, we evaluated the expression of GABA-Aα5 mRNA by RT-PCR. The results of RT-PCR revealed that the levels of GABA-Aα5 mRNA in the PFC of the male and female alcohol-exposed groups were significantly higher than those in the PFC of the normal control group (*P* < 0.05; Figure 3). In contrast, there was no significant difference in the expression level of GABA-Aα5 in the PFC between the male alcohol exposure group and the female alcohol exposure group (*P* > 0.05; Figure 3).

### Chronic alcohol treatment increases GABA-Aα5 histone H3K9 acetylation and H3K4 trimethylation in the PFC

**Figure 4:**
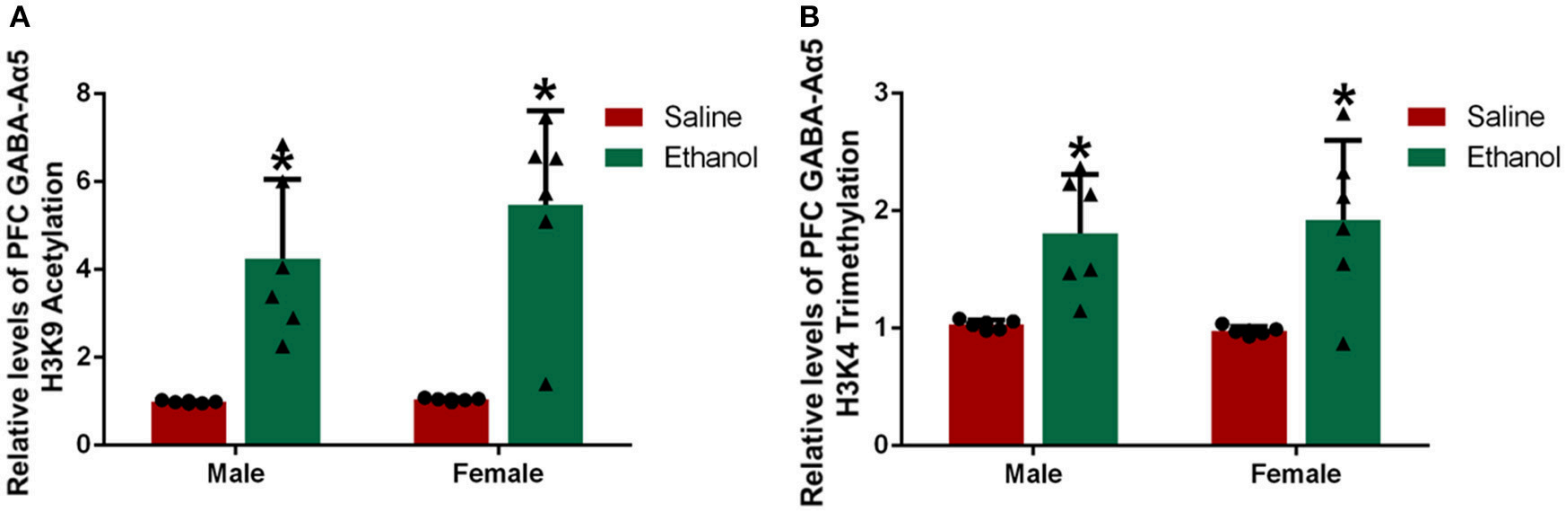
Chronic alcohol treatment increased GABA-Aα5 histone H3K9 acetylation and H3K4 trimethylation in the PFC. ChIP-PCR was used to detect the relative level of GABA-Aα5 histone H3K9 acetylation and H3K4 trimethylation. **(A)** The relative level of GABA-Aα5 histone H3K9 acetylation in the PFC. **(B)** The relative level of GABA-Aα5 histone H3K4 trimethylation in the PFC. The data are expressed as the mean ± SD (*n* = 6). *p < 0.05 vs. Saline, two-way ANOVA.

Histone H3K9 acetylation and H3K4 trimethylation are considered as important regulatory pathways for gene expression (12, 13). To explore the mechanism leading to the increased GABA-Aα5 mRNA levels caused by chronic alcohol treatment, we measured the level of GABA-Aα5 histone H3K9 acetylation and H3K4 trimethylation in the PFC by ChIP-PCR (Figures 4A,B). In addition, the levels of PFC GABA-Aα5 histone H3K9 acetylation and H3K4 trimethylation in the male and female alcohol-exposed groups were significantly higher than those in the normal control group (*P* < 0.05), indicating that the epigenetic changes caused by chronic alcohol treatment may also account for the increase in GABA-Aα5 mRNA (*P* < 0.05).

### Increased PFC GABA-Aα5 histone H3K4 trimethylation caused by chronic alcohol treatment can be inherited by the offspring

**Figure 5:**
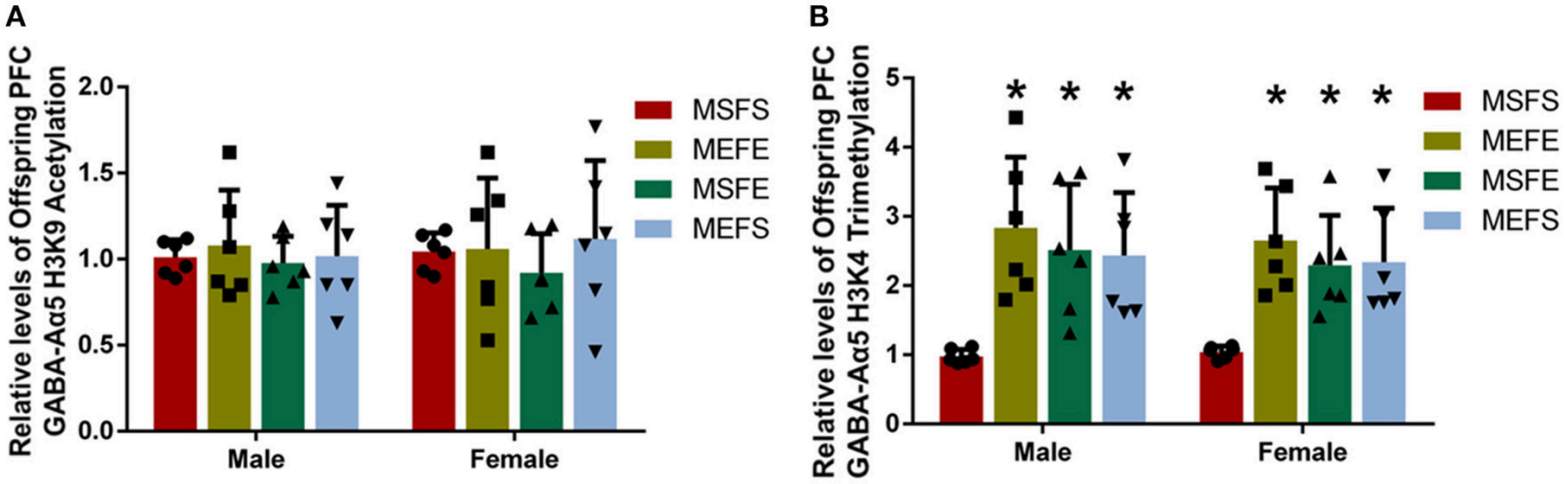
PFC GABA-Aα5 histone H3K4 trimethylation increased in offspring with an alcohol genetic background. ChIP-PCR was used to detect the relative level of GABA-Aα5 histone H3K9 acetylation and H3K4 trimethylation in the PFC of offspring. **(A)** The relative level of GABA-Aα5 histone H3K9 acetylation in the PFC of offspring. **(B)** The relative level of GABA-Aα5 histone H3K4 trimethylation in the PFC of offspring. The data are expressed as the mean ± SD (*n* = 6). ^*^p < 0.05 vs. MSFS, two-way ANOVA.

Partial epigenetic changes can be inherited by the offspring (14). To investigate whether the increases in PFC GABA-Aα5 histone H3K9 acetylation and H3K4 trimethylation caused by chronic alcohol treatment can be inherited by the offspring, we used rats for breeding after chronic alcohol treatment and then measured PFC GABA-Aα5 histone H3K9 acetylation and H3K4 trimethylation during adulthood (2 months). The results showed no significant difference between the levels of H3K9 acetylation in all eight subgroups (Figure 5A), whereas the levels of H3K4 trimethylation in the PE, FE, and ME subgroups were significantly higher than those in the male and female PS subgroups (*P* < 0.05; Figure 5B).

### PFC GABA-Aα5 mRNA is elevated in offspring with an alcohol genetic background

**Figure 6:**
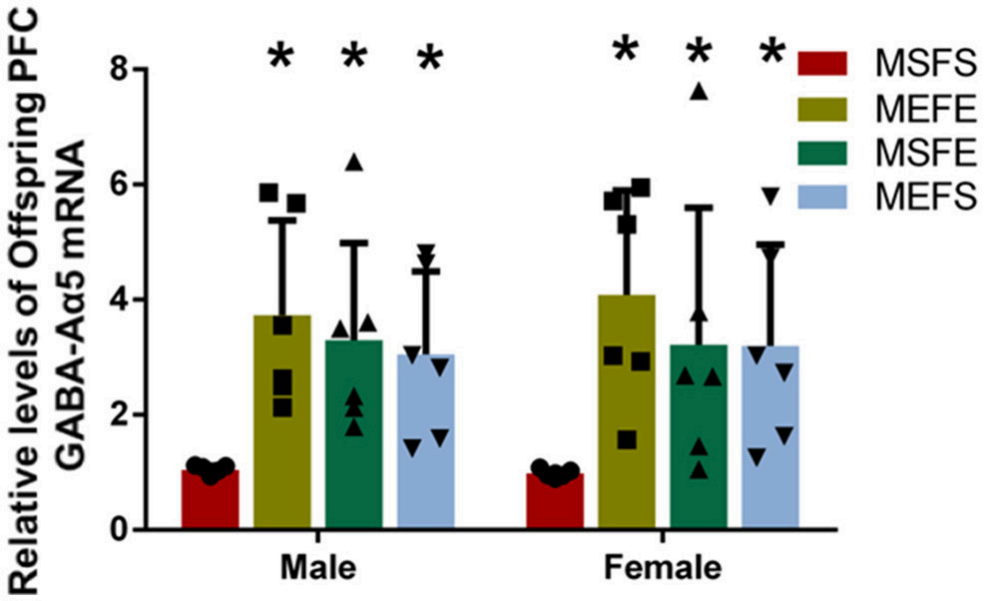
PFC GABA-Aα5 mRNA levels increased in offspring with an alcohol genetic background. The relative level of GABA-Aα5 mRNA in the PFC of offspring was detected by RT-PCR. The data are expressed as the mean ± SD (*n* = 6). ^*^p < 0.05 vs. FSMS, two-way ANOVA.

To investigate whether the increase in PFC GABA-Aα5 histone H3K4 trimethylation caused by an alcohol genetic background can induce PFC GABA-Aα5 expression, we detected the level of PFC GABA-Aα5 mRNA in offspring by RT-PCR (Figure 6). In male offspring, the expression of PFC GABA-Aα5 in the MEFE, MEFS, and MSFE groups was significantly higher than that in the MSFS group (*P* < 0.05). Similarly, in female offspring, the expression of PFC GABA-Aα5 mRNA in the MEFE, MEFS, and MSFE groups was significantly higher than that in the MSFS group (*P* < 0.05).

### Vulnerability to alcohol addiction is induced in offspring with an alcohol genetic background

**Figure 7:**
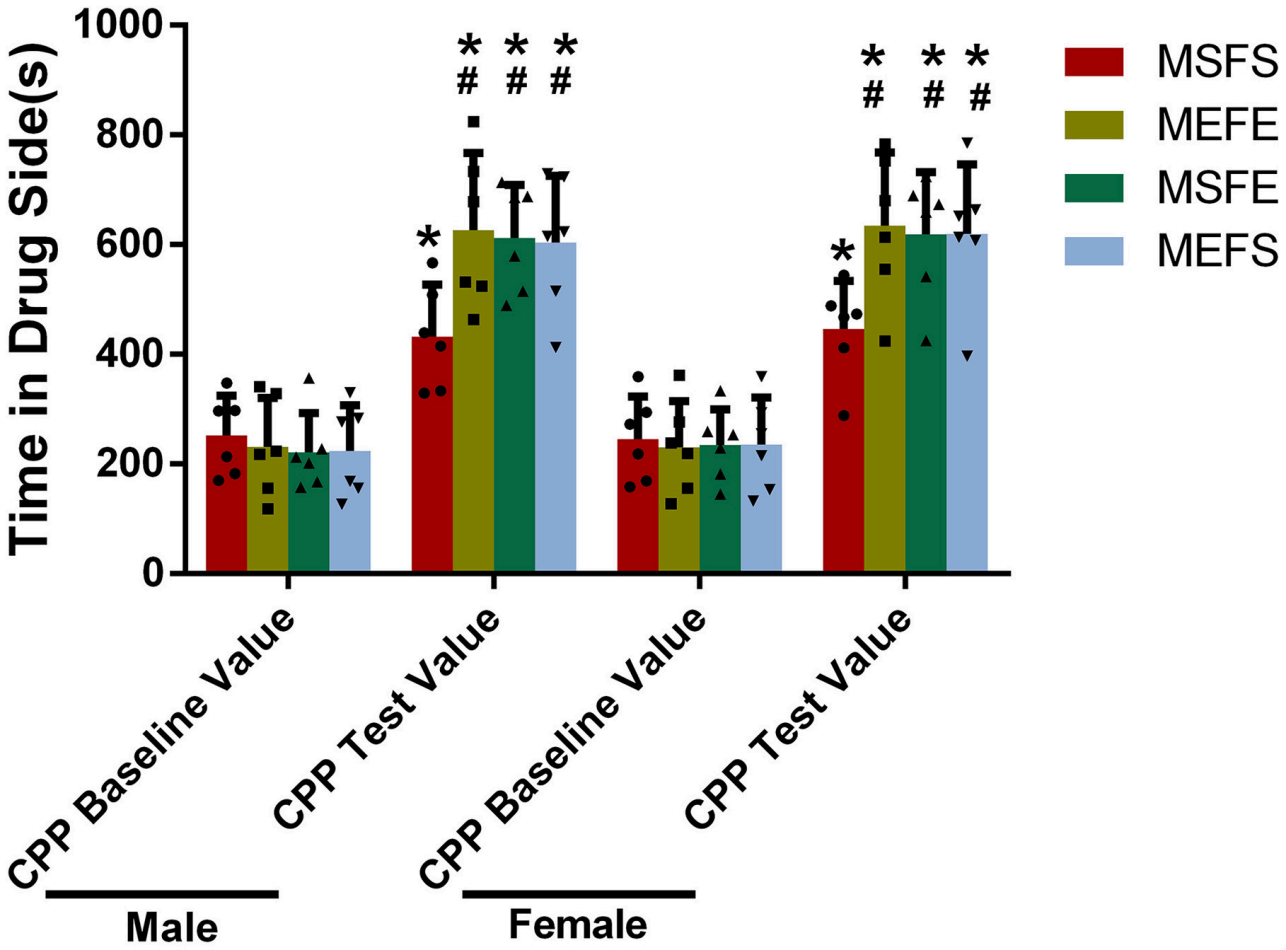
CPP test value increased in offspring with an alcohol genetic background. The offspring were treated with chronic alcohol during the training period and alcohol-related CPP was measured. CPP baseline value and CPP test value were recorded. The data are expressed as the mean ± SD (*n* = 6). ^*^p < 0.05 vs. CPP baseline value, ^#^*p* < 0.05 vs. MSFS, two-way ANOVA.

To investigate whether the offspring with an alcohol genetic background were more vulnerable to alcohol addiction, we treated them with chronic alcohol during the training period and measured alcohol-related CPP (Figure 7). All the groups had significantly higher CPP test values than CPP baseline values (*P* > 0.05), whereas no difference in CPP baseline values was found between all the groups. The MEFE, MEFS, and MSFE groups had significantly higher CPP test values than the male and female rats in the MSFS group (*P* < 0.05). Again, sex did not show any influence as no significant difference was found between the offspring of different sexes from the same treated parents. Additionally, there was no significant difference between the MEFE, MEFS, and MSFE groups (*P* > 0.05). This indicates that offspring with an alcohol genetic background have an increased vulnerability to alcohol addiction.

## Discussion

Studying biochemical mechanisms such as neurotransmitters, receptors, and signal transduction systems has been one of the focuses of research on drug addiction, including alcohol addiction (15–18). The relationship between GABA-A and alcohol addiction has always been appreciated. Evidence shows that GABA-A knockout mice have a significant reduction in alcohol consumption (double bottle method) (19). The frequency of GABA-A receptor-mediated inhibitory post-synaptic currents was depressed in the medium spiny neurons of ethanol drinking mice (20). It was reported that GABA-A/Glu coadjust DA to regulate alcohol addiction (21). In Wistar rats, GABA-Aα5 is a GABA-A receptor subunit that is positively correlated with spontaneous alcohol consumption (22). However, the relationship between GABA-Aα5 and alcohol addiction is currently unclear.

In this study, after 15 days of chronic alcohol treatment, the CPP test values in the male and female alcohol exposure groups were significantly higher than the CPP baseline values. In contrast, there was no significant difference between the test values and the CPP baseline values in the groups treated with saline, suggesting that alcohol exposure results in the formation of an alcohol reward memory in rats. Furthermore, PFC GABA-Aα5 mRNA levels were significantly higher in the male and female alcohol-exposed animals than in the control animals, indicating that chronic alcohol treatment might increase PFC GABA-Aα5 expression. This finding suggests that increased expression of PFC GABA-Aα5 may result from alcohol treatment.

Epigenetic modulation is an important way to regulate protein expression. Drug addiction can cause many epigenetic changes (23). In our study, both GABA-Aα5 histone H3K9 acetylation and H3K4 trimethylation increased in the PFC after chronic alcohol treatment, suggesting that chronic alcohol treatment might increase GABA-Aα5 expression through the upregulation of histone H3K9 acetylation and H3K4 trimethylation.

In the case of addiction, the epigenetic mechanism regulated by environmental stimuli can make individuals adapt to dynamic changes in the surrounding environment; this mechanism can also be partially inherited by future generations (24). Qiumin Le et al. [2017] found that cocaine treatment causes increased susceptibility to cocaine addiction in offspring (25). Our study also showed that PFC GABA-Aα5 mRNA and its histone H3K4 trimethylation were elevated in offspring with an alcohol genetic background. Simultaneously, the vulnerability to alcohol addiction was induced in offspring with an alcohol genetic background. These findings further suggest that GABA-Aα5 and its histone H3K4 trimethylation may participate in the regulation of the vulnerability to alcohol addiction and indicate that long-term alcoholism will make future generations more likely to be addicted to alcohol. This result may partly explain the following interesting phenomenon: if parents have moderate or high alcohol consumption, then their children are more likely to drink (26).

In conclusion, this study demonstrated that chronic alcohol treatment increases the expression of PFC GABA-Aα5 by inducing its histone H3K4 trimethylation. These changes could be hereditary and might be one of the reasons for increased vulnerability to alcohol addiction in offspring. If this conjecture is confirmed, PFC GABA-Aα5 could be a molecular target for prophylactic or curative treatment of alcohol addiction, providing new clues for drug development. However, this area of study is still in its infancy as the specific molecular mechanisms underlying the involvement of GABA-Aα5 in the formation of alcohol addiction are unclear. Additionally, whether inhibition of GABA-Aα5 expression will reduce alcohol addiction in long-term drinkers or if the difference in GABA-Aα5 expression alone will affect the susceptibility of different individuals to alcohol has not yet been elucidated. Furthermore, the mechanisms by which parental and offspring GABA-Aα5 expression in the PFC is increased by chronic alcohol treatment remain to be studied.

## Data accessibility

The raw data of the current study can be obtained from the corresponding author XL.

## Ethics statement

This study was carried out in accordance with the recommendations of Wuhan Mental Health Center Ethics Committee. The protocol was approved by the Wuhan Mental Health Center Ethics Committee.

## Author contributions

XL and WH designed the study and provided most of the funds. KZ and AX supervised the study and wrote the manuscript. XZ analyzed the data. BZ analyzed and interpreted the data, revised the manuscript and provided part of the funds.

### Conflict of interest statement

The authors declare that the research was conducted in the absence of any commercial or financial relationships that could be construed as a potential conflict of interest.
